# Hepatitis B Prevalence, Immune Status, and Persistence of Protection Among Dental Practitioners and Students: An Institutional Cross-Sectional Study

**DOI:** 10.7759/cureus.109828

**Published:** 2026-05-28

**Authors:** Shashikala Ramaswamy, Sanpreet S Sachdev, Karishma Ashok, Minal Kshirsagar, Dipooja Patil, Ashwini Panchmahalkar

**Affiliations:** 1 General Pathology and Microbiology, Bharati Vidyapeeth (Deemed to be University) Dental College and Hospital, Navi Mumbai, IND; 2 Oral Pathology and Microbiology, Bharati Vidyapeeth (Deemed to be University) Dental College and Hospital, Navi Mumbai, IND; 3 Periodontics, Bharati Vidyapeeth (Deemed to be University) Dental College and Hospital, Navi Mumbai, IND; 4 Public Health Dentistry, Bharati Vidyapeeth (Deemed to be University) Dental College and Hospital, Navi Mumbai, IND; 5 Conservative Dentistry and Endodontics, Bharati Vidyapeeth (Deemed to be University) Dental College and Hospital, Navi Mumbai, IND

**Keywords:** anti-hbc, dental professionals, hepatitis b, needle stick injury, occupational exposure, seroprotection

## Abstract

Background

Dental professionals are at increased risk of occupational exposure to the hepatitis B virus (HBV) because of frequent contact with blood, saliva, and sharp instruments. In addition to exposure history, assessment of self-reported vaccination history, serological immune status, and markers of prior exposure is important for understanding the occupational risk profile.

Aim

This study aimed to assess HBV-related knowledge, occupational exposure, self-reported vaccination history, serological immune status, and antibody to hepatitis B core antigen (anti-HBc) reactivity among dental professionals in a teaching institution.

Methods

This single-center cross-sectional observational study was conducted among undergraduate students, postgraduate students, interns, and faculty members in a dental college and hospital in Navi Mumbai. Of 500 participants, 436 completed the questionnaire and were included in the final analysis. The structured questionnaire assessed knowledge of HBV transmission, prevention, post-exposure prophylaxis, vaccination history, and occupational exposure history. Blood samples were voluntarily obtained from 275 consenting participants and analyzed for antibody to hepatitis B surface antigen (anti-HBs), anti-HBc, and hepatitis B surface antigen (HBsAg).

Results

Overall knowledge regarding HBV transmission, prevention, and post-exposure prophylaxis was high. Needle stick injury was reported by 14.0% of participants, and 17.2% reported mucocutaneous exposure to blood or body fluids. Among those tested, 55.6% had anti-HBs titers of >100 mIU/mL, 14.9% had titers of 10-100 mIU/mL, and 29.5% had titers of <10 mIU/mL. Anti-HBc reactivity was observed in 10.9% of tested participants, while no participant was HBsAg positive. Anti-HBc reactivity was significantly more frequent among participants with a history of needle stick injury and mucocutaneous exposure.

Conclusion

Good HBV-related knowledge coexisted with meaningful occupational exposure and a substantial subgroup with non-protective anti-HBs levels. These findings support a stronger institutional emphasis on exposure prevention, documented vaccination completion, and verification of immune status in dental professionals.

## Introduction

Hepatitis B virus (HBV) infection remains a major public health problem worldwide because of its high burden of chronic liver disease, cirrhosis, and hepatocellular carcinoma [1]. Among healthcare workers, HBV is especially important because occupational exposure to blood and body fluids continues to occur despite advances in vaccination and infection-control protocols. Percutaneous injury, contact with contaminated sharp instruments, and improper post-exposure management remain recognized routes of occupational risk [2,3]. Healthcare professionals are therefore a priority group for hepatitis B prevention, vaccination, and serological surveillance.

Dental professionals represent a particularly vulnerable subgroup of healthcare workers. Routine dental procedures involve frequent handling of needles, burs, scalpels, and other sharp instruments in a blood- and saliva-contaminated environment [4]. These procedures are performed in close proximity to the patient's oral cavity, increasing the likelihood of contact with potentially infectious material. Generation of aerosols in the limited working space of dental clinics during procedures such as ultrasonic scaling or cavity preparation with airotors is another concern for occupational safety [5]. For this reason, dentistry has long been recognized as an occupation with potential exposure to blood-borne pathogens, particularly HBV and human immunodeficiency virus (HIV) [6,7]. Although hepatitis B vaccination is highly effective, institutional protection depends not only on vaccine uptake but also on the completion of the schedule, persistence of protective antibody levels, adherence to standard precautions, awareness of post-exposure prophylaxis, and timely reporting of exposure incidents [2].

Previous studies from India and other settings have examined awareness, preventive practices, vaccination coverage, and needle stick injuries (NSI) among dental students and dental professionals. These studies generally report moderate to good awareness, but they also highlight persistent deficiencies in vaccine completion, exposure reporting, and practical knowledge of post-exposure management [8-10]. However, much of the available literature is questionnaire-based and often limited to students or selected subgroups. Few studies have combined questionnaire-based assessment of knowledge and occupational exposure history with objective serological markers within the same dental teaching environment. As a result, much of the existing literature cannot distinguish between perceived preparedness, vaccine-linked immune status, and serological evidence of previous HBV exposure. Inclusion of antibody to hepatitis B core antigen (anti-HBc) testing is particularly important in this context because it adds evidence of past exposure that cannot be inferred from knowledge scores, self-reported vaccination history, or antibody to hepatitis B surface antigen (anti-HBs) titers alone. This combined approach is relevant to institutional surveillance because it links reported occupational risk with measurable biological markers in the same population.

Against this background, the present study was conducted among undergraduate and postgraduate students, interns, and faculty members at a dental teaching institution in Navi Mumbai. The aim of this single-center, cross-sectional study was to assess hepatitis B-related occupational risk and serological status among dental professionals. The specific objectives were to evaluate knowledge regarding HBV transmission, prevention, and post-exposure prophylaxis, document the occurrence of NSI and unprotected mucocutaneous exposure, and assess serological evidence of protection and past exposure using serological markers. By integrating questionnaire findings with anti-HBs, anti-HBc, and hepatitis B surface antigen (HBsAg) testing in a single-center dental cohort, the study was also intended to generate evidence relevant to institutional HBV surveillance and occupational health monitoring in dental training settings.

## Materials and methods

The present cross-sectional observational study was conducted over a period of two years, from January 2023 to January 2025, at YMT Dental College and Hospital, Navi Mumbai, India. The study population comprised a total of 500 undergraduate dental students, postgraduate students, interns, and teaching faculty from all nine departments of the institution.

Ethical approval for the study was obtained from the Institutional Ethics Committee of YMT Dental College and Hospital (approval number: YMTDC/IEC/OUT/160; dated 03/06/2022). Written informed consent was obtained from all participants prior to enrolment, and participant anonymity was maintained throughout the study. The study was designed not only to assess knowledge and self-reported occupational exposure but also to objectively determine immune protection and previous exposure to HBV using serological markers. Therefore, blood sampling was ethically justified as these outcomes could not be established through questionnaire data alone. Blood collection was performed only in participants who consented to serological testing. All staff members and students who provided written informed consent were considered eligible for participation. Of the 500 volunteers, 436 participants submitted fully completed questionnaires and were included in the questionnaire-based analysis.

Sample size calculation

The sample size for this cross-sectional study was calculated using the standard formula for estimating a single proportion in prevalence studies, n = Z²pq/d², where n is the required sample size, Z is the standard normal deviate at 95% confidence (1.96), p is the expected proportion, q = 1 − p, and d is the absolute precision. As no prior institutional estimate was available, the expected proportion was taken from the study by Kumar et al., who reported hepatitis B vaccination coverage of 44.4% among Indian dental students [9]. Using p = 0.444, q = 0.556, and d = 0.05, the minimum required sample size was 379.3, rounded to 380. After allowing for a 10% non-response rate, the final minimum sample size was calculated to be 417.3. After rounding off, it was discerned that 418 participants would suffice to fulfil the objectives of the present study.

Study procedure and data collection

The study was carried out in two phases. In the first phase, hepatitis B-related knowledge, vaccination status, and self-reported occupational exposure history were assessed using a structured questionnaire (see Appendices). The questionnaire captured information on vaccination status and occupational risk-related variables, organized under the domains of HBV knowledge, reported vaccination history, and self-reported occupational exposure, including knowledge of HBV transmission in the dental setting, the preventive role of vaccination and infection-control measures, awareness of post-exposure prophylaxis, history of NSI, and history of unprotected mucocutaneous exposure to blood or body fluids. The structured questionnaire was self-constructed for the present study with reference to the study objectives. Content validity was assessed by a panel of five senior faculty experts from the institution, who reviewed the questionnaire for relevance, clarity, sequence, and adequacy of coverage of the intended domains. Based on their suggestions, revisions were made to the wording, framing, and ordering of several items. The revised questionnaire was then pilot-tested in 15 participants drawn from the institutional population but not included in the final analysis, in order to assess clarity, acceptability, and internal consistency. The overall Cronbach's alpha for the questionnaire was 0.82, indicating good internal consistency. 

Blood sample collection and serological assessment

Among the 436 participants who completed the questionnaire, 275 consented to blood collection for serological investigation in order to objectively assess anti-HBs, anti-HBc, and HBsAg status. Participation in serological testing was voluntary and based on separate consent. Blood samples were collected on four different days and analyzed for hepatitis B immune status. Anti-HBs and anti-HBc were assessed using chemiluminescence immunoassay (CLIA), and HBsAg was determined by rapid card testing. The immune assay was performed using the AutoLumo A1000 fully automated CLIA analyzer (MatrixLabs, Chennai, India), which is based on a microparticle chemiluminescence platform for qualitative and quantitative biomarker detection.

Definitions of serological outcomes

Serum anti-HBs levels were used to categorize immune status against hepatitis B. For descriptive analysis, anti-HBs titers among seroprotected participants were further stratified into 10-100 mIU/mL and >100 mIU/mL, consistent with older serological literature that has used >100 IU/L as a higher-response category [11]. However, guideline-defined seroprotection was considered at anti-HBs of ≥10 mIU/mL. Anti-HBc reactivity was interpreted as evidence of previous exposure to HBV. Samples requiring further evaluation were also tested for HBsAg to identify current infection status.

Outcome measures

The primary outcome measures were the prevalence of self-reported NSI, the prevalence of unprotected mucocutaneous exposure, vaccination status, hepatitis B-related knowledge indicators, seroprotection based on anti-HBs titers, and anti-HBc reactivity as a marker of past exposure. Analyses were also performed according to professional category and department.

Statistical analysis

Data were summarized primarily as frequencies and percentages. Group-wise comparisons of categorical variables, including occupational exposures, questionnaire responses, blood sample collection, immune-status distribution, and anti-HBc reactivity, were performed using the Pearson chi-squared test and the Z test as reported in the study data. Odds ratios with 95% confidence intervals were calculated for selected 2 × 2 associations. To further explore factors associated with previous HBV exposure, a binary logistic regression model was fitted with anti-HBc reactivity as the dependent variable. Occupational exposure variables, including history of NSI and mucocutaneous blood/body fluid exposure, were entered as predictors in the adjusted model. Adjusted odds ratios with 95% confidence intervals were calculated. A p-value of less than 0.05 was considered statistically significant.

## Results

A total of 500 dental professionals and students volunteered for the study, of whom 436 submitted completed questionnaires and were included in the final analysis. Most respondents were aged 19-30 years (88.8%) and were female (74.5%). Undergraduate students formed the largest subgroup, followed by postgraduate students, interns, and faculty members. This age distribution reflects the institutional composition of the study sample rather than the age structure of the wider dental workforce. Nearly half of the participants had no independent clinical exposure, whereas 26.4% had less than five years of clinical exposure. A history of NSI was reported by 61 participants (14.0%), and 75 participants (17.2%) reported unprotected mucocutaneous exposure to blood or body fluids. Blood samples for serological investigation were obtained from 275 participants (63.1%). As serological testing was performed only in participants who consented to blood sampling, the serological findings should be interpreted as applying to this tested subgroup rather than to the entire questionnaire cohort.

**Figure 1:**
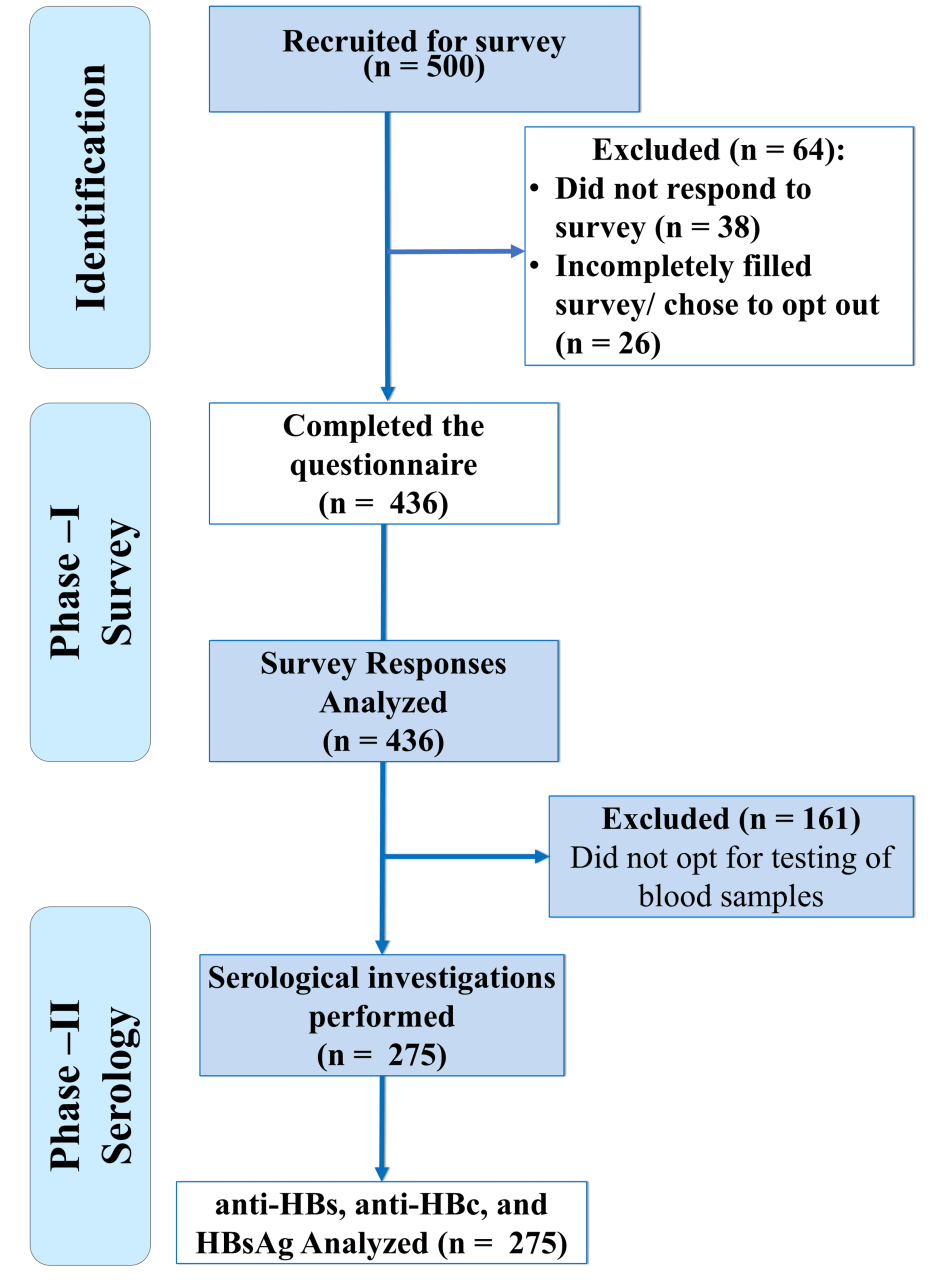
STROBE flow diagram delineating the study process from recruitment to survey to serological analysis STROBE: Strengthening the Reporting of Observational Studies in Epidemiology; anti-HBs: antibody to hepatitis B surface antigen; anti-HBc: antibody to hepatitis B core antigen; HBsAg: hepatitis B surface antigen

The study process from recruitment to survey to serological investigations is delineated as a flow diagram in Figure 1. Among those tested, 153 (55.6%) were fully protected against hepatitis B, 41 (14.9%) were partially protected, and 81 (29.5%) were unprotected. Anti-HBc reactivity was reported in 30 participants (10.9%), and none of the reactive samples were HBsAg positive (Table 1). Because participation in serological testing was voluntary, differences between tested and non-tested participants cannot be excluded, and the observed prevalence of anti-HBs and anti-HBc markers may not fully represent the entire study population.

**Table 1:** Baseline characteristics, occupational exposure, and serological profile of the study participants Anti-HBs: antibody to hepatitis B surface antigen; anti-HBc: antibody to hepatitis B core antigen; HBsAg: hepatitis B surface antigen

Variable	n (%)
Questionnaire respondents	436 (100.0)
Age 19-30 years	387 (88.8)
Age 31-40 years	30 (6.9)
Age >40 years	19 (4.4)
Female	325 (74.5)
Male	111 (25.5)
Undergraduate students	250 (57.3)
Postgraduate students	68 (15.6)
Interns	64 (14.7)
Faculty members	54 (12.4)
No clinical exposure	215 (49.3)
<5 years of clinical exposure	115 (26.4)
5-10 years of clinical exposure	68 (15.6)
11-15 years of clinical exposure	18 (4.1)
16-21 years of clinical exposure	7 (1.6)
≥22 years of clinical exposure	13 (3.0)
Needle stick injury	61 (14.0)
Mucocutaneous blood/body fluid exposure	75 (17.2)
Participants tested serologically	275 (63.1)
Fully protected (anti-HBs >100 mIU/mL)	153 (55.6)
Partially protected (anti-HBs 10-100 mIU/mL)	41 (14.9)
Unprotected (anti-HBs <10 mIU/mL)	81 (29.5)
Anti-HBc reactive	30 (10.9)
HBsAg positive	0 (0.0)

Overall knowledge regarding hepatitis B was good across most questionnaire domains. Favorable responses exceeded 94% for occupational risk, transmission in the dental setting, prevention by vaccination and infection-control practices, and post-exposure prophylaxis. Knowledge was comparatively lower for the item concerning HBV survival outside the body for at least seven days, for which only 69.1% gave a favorable response and 29.7% remained uncertain. The proportion of unfavorable responses was low across all items (Table 2).

**Table 2:** Item-wise knowledge regarding hepatitis B among questionnaire respondents Favorable response: strongly agree + agree; unfavorable response: disagree + strongly disagree; NSI: needle stick injury; HBIG: hepatitis B immunoglobulin

Knowledge item	n	Favorable response, n (%)	Uncertain, n (%)	Unfavorable response, n (%)
Dentists are at high occupational risk for hepatitis B	436	417 (95.6)	19 (4.4)	0 (0.0)
Hepatitis B infection may be asymptomatic in many cases	435	388 (89.2)	47 (10.8)	0 (0.0)
Hepatitis B may progress to chronic hepatitis, cirrhosis, or liver cancer	436	388 (89.0)	45 (10.3)	3 (0.7)
Hepatitis B is transmitted through blood and sexual, vertical, and household routes	436	424 (97.2)	11 (2.5)	1 (0.2)
Hepatitis B can be transmitted in the dental setting through blood/saliva exposure and NSI	436	418 (95.9)	13 (3.0)	5 (1.1)
Hepatitis B can be prevented by vaccination and infection-control measures	436	421 (96.6)	14 (3.2)	1 (0.2)
Exposed persons should report and receive post-exposure prophylaxis with HBIG	436	413 (94.7)	21 (4.8)	2 (0.5)
Hepatitis B virus can survive outside the body for at least 7 days	434	300 (69.1)	129 (29.7)	5 (1.2)

Profession-wise analysis showed significant differences in occupational exposure and immune profile (Table 3). NSI was highest among faculty members (35.2%) and postgraduate students (27.9%), and the difference across professional groups was significant (p < 0.001). Mucocutaneous exposure was most frequent among postgraduate students (39.7%) and faculty members (38.9%), and this difference was also significant (p < 0.001). Willingness to undergo blood testing differed across groups (p = 0.020), with the highest participation among faculty members (81.5%). Immune status also differed significantly by profession (p < 0.001). Faculty members showed the highest proportion of full protection (68.2%), whereas undergraduate students had the highest proportion of unprotected individuals (44.6%).

**Table 3:** Profession-wise distribution of occupational exposure and immune status The Pearson chi-squared test was used for profession-wise comparisons. *Percentages for immune-status categories were calculated out of the number of participants tested in each specialty. NSI: needle stick injury

Profession	Total respondents, n	NSI, n (%)	Mucocutaneous exposure, n (%)	Blood samples collected, n (%)	Fully protected*, n (%)	Partially protected*, n (%)	Unprotected*, n (%)
Undergraduate students	250	13 (5.2)	16 (6.4)	148 (59.2)	73 (49.3)	9 (6.1)	66 (44.6)
Postgraduate students	68	19 (27.9)	27 (39.7)	41 (60.3)	25 (61.0)	11 (26.8)	5 (12.2)
Interns	64	10 (15.6)	11 (17.2)	42 (65.6)	25 (59.5)	12 (28.6)	5 (11.9)
Faculty members	54	19 (35.2)	21 (38.9)	44 (81.5)	30 (68.2)	9 (20.5)	5 (11.4)
Total	436	61 (14.0)	75 (17.2)	275 (63.1)	153 (55.6)	41 (14.9)	81 (29.5)
Test statistic	-	χ² = 47.35	χ² = 62.49	χ² = 9.87	χ² = 45.38	χ² = 45.38	χ² = 45.38
P-value	-	<0.001	<0.001	0.020	<0.001	<0.001	<0.001

The anti-HBc reactivity was more frequent among participants with a history of occupational exposure. Participants with prior NSI showed a significantly higher proportion of anti-HBc reactivity than those without NSI (p = 0.001). Similarly, participants with a history of mucocutaneous exposure had significantly higher anti-HBc reactivity than those without such exposure (21.4% vs 8.7%; p = 0.016). Profession-wise variation in anti-HBc reactivity was not significant (p = 0.456) (Table 4). 

**Table 4:** Association of occupational exposure history with anti-HBc reactivity N=275; the Pearson chi-squared test was used for association analyses. Anti-HBc: antibody to hepatitis B core antigen; CI: confidence interval

Exposure variable	Anti-HBc reactive among exposed, n/N (%)	Anti-HBc reactive among unexposed, n/N (%)	Odds ratio (95% CI)	Test statistic	P-value
Needle stick injury	12/46 (26.1)	18/217 (8.3)	3.90 (1.73-8.82)	χ² = 10.19	0.001
Mucocutaneous blood/body fluid exposure	12/56 (21.4)	18/207 (8.7)	2.86 (1.29-6.38)	χ² = 5.87	0.016

To further assess these associations, binary logistic regression was performed with anti-HBc reactivity as the dependent variable (Table 5). The model was significant overall (likelihood-ratio χ² = 11.82; p = 0.003). After adjustment for the other occupational exposure variable, history of NSI remained significantly associated with anti-HBc reactivity (adjusted odds ratio: 3.11; 95% CI: 1.26-7.67; p = 0.014), whereas mucocutaneous exposure did not retain independent statistical significance (adjusted odds ratio: 1.84; 95% CI: 0.75-4.51; p = 0.182).

**Table 5:** Binary logistic regression analysis for predictors of anti-HBc reactivity Predictors entered into the model were history of needle stick injury and history of mucocutaneous blood/body fluid exposure. Analyses were based on participants with available analyzable anti-HBc entries (n = 263). Anti-HBc: antibody to hepatitis B core antigen; CI: confidence interval

Predictor	Adjusted odds ratio	95% CI	P-value
Needle stick injury	3.11	1.26-7.67	0.014
Mucocutaneous blood/body fluid exposure	1.84	0.75-4.51	0.182

## Discussion

The present study provides an integrated assessment of hepatitis B-related knowledge, occupational exposure, and serological evidence of past exposure among dental professionals in a teaching institution. This combined design adds an important dimension to the existing literature because it moves beyond questionnaire-only assessment and allows the simultaneous evaluation of reported occupational risk, measurable immune status, and serological evidence of previous exposure. In particular, anti-HBc testing strengthens the interpretation of occupational risk by identifying prior HBV exposure that would not be captured by self-reported vaccination history or anti-HBs measurement alone. Overall awareness was high, yet clinically relevant exposure remained common, and a substantial subgroup of tested participants lacked seroprotective anti-HBs levels. A plausible explanation is that knowledge alone does not adequately translate into effective occupational protection when exposure-prone clinical work, incomplete vaccination documentation, and absence of immune-status verification coexist within the same institutional environment. This pattern is important because occupational protection against hepatitis B depends not only on awareness but also on the completion of the vaccine schedule, documentation of protective antibody response, adherence to standard precautions, and timely post-exposure management after occupational incidents [12]. 

The favorable knowledge profile observed in this study is broadly consistent with previous Indian dental literature. Mahesh et al. reported good awareness regarding hepatitis B among dental graduate students, while Kumar et al. found acceptable knowledge and opinion scores but less satisfactory preventive practices among Indian dental students [8,9]. Taken together, these findings suggest that awareness in dental settings may be necessary but not sufficient to reduce occupational vulnerability, particularly when infection-control knowledge is uneven across domains. The relatively weaker understanding of environmental persistence of HBV may reflect emphasis on general transmission teaching without equal reinforcement of practical environmental-risk concepts, which are directly relevant to surface contamination, sharps handling, and post-procedure operatory safety. This is clinically relevant because HBV can remain viable on contaminated surfaces for at least seven days, making environmental contamination and procedural lapses important from an infection-control perspective [12].

The occupational exposure burden in the present study is also in keeping with previous dental literature. Pavithran et al. documented a substantial burden of NSI and sharps injury among dental professionals in Bangalore, with gaps in reporting and post-exposure practices, while Wicker and Rabenau demonstrated that occupational exposure to blood and body fluids remains common among dental professionals and students in clinical environments [7,10]. The higher frequencies of NSI and mucocutaneous exposure among postgraduate students and faculty members in the present study are biologically plausible, as these groups are more intensively involved in invasive, restorative, surgical, and needle-based procedures and therefore have greater cumulative exposure to blood-contaminated instruments and body fluids. This distribution likely reflects a gradient of procedural intensity rather than a simple gradient of knowledge, with greater involvement in surgical, endodontic, prosthodontic, and needle-based procedures increasing the probability of cumulative exposure over time. The interpretation is supported by infection-control literature that recognizes dentistry as a setting with recurring risk of exposure to bloodborne pathogens if barrier precautions, sharps handling, and procedural safeguards are not consistently maintained [13,14].

The serological findings add practical depth to the questionnaire-based observations. Although awareness of vaccination was high, a meaningful proportion of tested participants had anti-HBs levels below the accepted seroprotective threshold. This is consistent with previous reports showing that measurable antibody protection may be inadequate in a subset of vaccinated trainees and healthcare workers. Lingawi and Afifi reported that a considerable proportion of dental students lacked seroprotective anti-HBs titers two decades after infant immunization, suggesting that measurable protection may be insufficient at the start of clinical training [15]. Similarly, Sahana et al. observed declining anti-HBs levels over time among medical students and healthcare workers, particularly with increasing time since vaccination [16]. Earlier work by Barash et al. also found that a notable proportion of vaccinated healthcare workers lacked serologic evidence of hepatitis B immunity, supporting the value of post-vaccination antibody verification in occupationally exposed groups [17]. One likely explanation is that vaccination history in institutional settings may not always be accompanied by documented post-vaccination antibody verification, so individuals may be assumed to be protected despite absent or waning measurable titers. From an institutional surveillance perspective, these findings suggest that monitoring strategies based only on awareness or self-reported vaccination history may be insufficient and that documentation of vaccine completion, structured exposure reporting, and targeted serological verification may provide a more informative assessment of occupational HBV risk. However, because detailed vaccination records were not uniformly available in the present study, these findings should be interpreted as reflecting serological immune status in relation to reported vaccination history rather than as a precise analysis of vaccine-response patterns.

At the same time, the present findings should not be interpreted to mean that declining or undetectable circulating antibody always indicates the absence of immune memory. Dini et al. demonstrated persistence of protection and an anamnestic response in healthcare students many years after primary immunization, while Cocchio et al. reported long-term persistence of anti-HBs in healthcare workers, particularly in those with stronger initial responses [18,19]. Therefore, the present findings are best interpreted as indicating that a subgroup of dental personnel may require verification of immune status rather than assuming adequate protection solely on the basis of self-reported vaccination history. In the same way, the inclusion of anti-HBc allows the study to move beyond vaccine-response assessment alone and to identify participants with serological evidence of previous exposure, thereby providing a broader picture of occupational HBV surveillance than anti-HBs assessment alone in dental institutions. The association observed between anti-HBc reactivity and histories of NSI and mucocutaneous exposure is noteworthy, but it should be interpreted cautiously. In the adjusted model, NSI remained independently associated with anti-HBc reactivity, which supports the relevance of percutaneous occupational exposure in this setting, although the cross-sectional design still precludes causal inference. Because of the cross-sectional design, these findings indicate association rather than causation, and they do not establish the exact timing, route, or setting of prior HBV exposure. They should therefore not be taken as proof of direct workplace transmission.

This study has several limitations. It was conducted at a single dental teaching institution; therefore, the findings may not be generalizable to other institutions or to the wider population of dental practitioners. The study population was weighted toward undergraduate students and trainees, resulting in a predominantly younger age distribution that reflects the institutional sample rather than the broader dental workforce. Occupational exposure history was self-reported and may have been affected by recall bias or underreporting. In addition, vaccination history was not characterized in sufficient detail to permit a more refined interpretation of immune status. Information such as the documented number of doses received, timing since vaccination, booster doses, and distinction between childhood and adult vaccination was not uniformly available, which limits the extent to which anti-HBs findings can be linked to specific vaccination patterns. Serological testing was available only in the subgroup that consented to blood sampling, which introduces an important possibility of selection bias. Participants who agreed to blood testing may have differed systematically from those who declined, for example, by being more health conscious, more aware of occupational risk, more compliant with vaccination, or alternatively more concerned about prior exposure. As a result, the observed prevalence of non-protective anti-HBs levels and anti-HBc reactivity may not accurately represent the full questionnaire cohort, and these serological findings should therefore be interpreted with caution. This limitation affects the external validity of the serological estimates and may also influence the strength of the observed associations if the tested subgroup differed meaningfully from the non-tested participants.

A formal comparison between tested and non-tested participants was not available, which further limits the assessment of the direction and magnitude of this potential bias. In addition, the anti-HBc association analysis was based on the subset with available analyzable anti-HBc entries rather than the entire serologically tested cohort. HBsAg screening was performed using a rapid card method, and confirmatory testing was not available; therefore, some degree of laboratory misclassification cannot be entirely excluded. Finally, anti-HBc reactivity indicates previous exposure to HBV but does not prove that exposure occurred specifically through an NSI or within the workplace. Taken together, these limitations mean that the present findings should be interpreted as hypothesis-generating evidence regarding the relationship between occupational exposure patterns, serological immune status alongside self-reported vaccination history, and previous HBV exposure in dental institutions. Future multicenter studies with larger and more diverse samples, verified vaccination records, complete post-vaccination antibody testing, confirmatory virological assessment where indicated, and prospective exposure surveillance would provide stronger evidence. Longitudinal studies would be particularly useful to clarify how clinical seniority, procedural workload, vaccine response, waning antibody levels, and post-exposure practices interact over time.

## Conclusions

Within the limits of this single-center cross-sectional study, good hepatitis B-related knowledge among dental professionals did not fully correspond with lower reported occupational exposure or uniformly protective antibody levels. The findings support stronger institutional emphasis on exposure prevention, documented vaccination completion, and verification of immune status in dental training and practice settings.

## Appendices

Survey questionnaire

PARTICIPANT CODE:­ NM/HBISD/2023/_______

1. Name: ________________________________________________________

2. Age

(a) 19-30 years

(b) 31-40 years

(c) More than 40 years

3. Gender

(a) Male

(b) Female

4. Date of birth: ________________________________________

5. Student or faculty

(a) I BDS

(b) II BDS

(c) III BDS

(d) IV BDS

(e) Internship

(f) I MDS

(g) II MDS

(h) III MDS

(i) Faculty

6. Working department (for faculty/PGs/interns)

(a) Department of Periodontics

(b) Department of Conservative Dentistry and Endodontics

(c) Department of Oral and Maxillofacial Surgery

(d) Department of Prosthodontics

(e) Department of Orthodontics

(f) Department of Pediatric Dentistry

(g) Department of Oral Pathology and Microbiology

(h) Department of Oral Medicine and Radiology

(i) Department of Public Health

7. Duration of work as a dental professional/clinical exposure to dental patients

(a) No exposure

(b) Less than 5 years

(c) 5-10 years

(d) 11-15 years

(e) 16-21 years

(f) 22 years and above

8. Among the healthcare professionals, dentists are placed in a high-risk group and are at the greatest occupational risk of hepatitis B infection

(a) Strongly agree

(b) Agree

(c) Uncertain

(d) Disagree

(e) Strongly disagree

9. All hepatitis B infections do not have symptoms. In the majority of cases, the infection may lead to acute hepatitis, which manifests as jaundice

(a) Strongly agree

(b) Agree

(c) Uncertain

(d) Disagree

(e) Strongly disagree

10. Few cases of hepatitis B infection progress to chronic hepatitis, leading to liver cirrhosis and liver cancer

(a) Strongly agree

(b) Agree

(c) Uncertain

(d) Disagree

(e) Strongly disagree

11. Hepatitis B infection is commonly transmitted through unscreened blood transfusion, through unprotected sexual contact, from mother to child, and also from close household contact, tattoo, and acupuncture practice

(a) Strongly agree

(b) Agree

(c) Uncertain

(d) Disagree

(e) Strongly disagree

12. In a dental setup, hepatitis B infection is transmitted through direct contact with blood, saliva, or nasopharyngeal droplets from infected patients/asymptomatic carriers during the dental procedure and also through needle stick injuries

(a) Strongly agree

(b) Agree

(c) Uncertain

(d) Disagree

(e) Strongly disagree

13. Hepatitis B infection can be prevented by vaccination and by Infection control guidelines at work

(a) Strongly agree

(b) Agree

(c) Uncertain

(d) Disagree

(e) Strongly disagree

14. Persons exposed to the risk of infection should report and get post-exposure prophylaxis with HBIG

(a) Strongly agree

(b) Agree

(c) Uncertain

(d) Disagree

(e) Strongly disagree

15. Hepatitis B virus can survive outside the body for at least 7 days. During that time, the virus is still capable of causing infection

(a) Strongly agree

(b) Agree

(c) Uncertain

(d) Disagree

(e) Strongly disagree

16. History of vaccination (year of last vaccination): _______________________________

17. Vaccination status

(a) Received one dose

(b) Received two doses

(c) Received three doses and booster doses

18. Complete vaccination status

(a) Fully vaccinated

(b) Partially vaccinated

(c) Not vaccinated

19. Reasons for not taking the vaccine, if not taken earlier

(a) I feel I'm protected

(b) Fear of side effects

(c) Did not give much emphasis

(d) The duration of the total dose is too long

(e) Not organized by the institution

20. Any history of exposure to a needle stick injury

(a) Yes

(b) No

21. Have you ever been exposed to unprotected mucocutaneous fluid/blood contact?

(a) Yes

(b) No

22. Have you ever been diagnosed with hepatitis B?

(a) Yes

(b) No

23. Have you received blood in the past?

(a) Yes

(b) No

24. Are you willing to know your hepatitis B immune status by providing a blood sample for a serological test?

(a) Yes

(b) No

Signature of the participant:                                                           Date:

………………………………………………………………………………….                                  (To be filled by the researcher only)

Blood sample ID:_______________________

Laboratory result:______________________

                              ______________________

                              ______________________
